# Metabolic profiling of prebiotic, probiotic, and synbiotic supplements in a canine colonic fermentation model: a pilot feasibility study reveals distinct and complementary effects

**DOI:** 10.3389/fvets.2025.1641809

**Published:** 2025-11-17

**Authors:** Alessandro Gramenzi, Luana Clerico, Benedetta Belà, Meri Di Leonardo, Isa Fusaro, Giulia Pignataro

**Affiliations:** 1 Department of Veterinery Medicine, University of Teramo, Teramo, Italy; 2 Independent Researcher, Savona, Italy; 3 Azienda Usl, Teramo, Italy

**Keywords:** canine gut microbiota, prebiotic, probiotic, metabolomics, colonic fermentation model, pilot feasibility study

## Abstract

**Introduction:**

The gut microbiota is a central player in canine health, influencing digestion, immune modulation, and metabolic homeostasis. Microbiota imbalance fuels interest in dietary interventions such as prebiotics, probiotics, and synbiotics (a combination of prebiotics and probiotics).

**Methods:**

This pilot feasibility study employed the SCIME™, a novel *in vitro* fermentation model simulating the canine colonic environment, to evaluate the metabolic effects of three dietary supplements using fecal inoculum from a single healthy canine donor. Products tested were Microbiotal (product M), a probiotic (product P, *Lactobacilli reuteri*), and a synbiotic (product M + P: Microbiotal *+L. reuteri*). Over a 2-week treatment period, fermentation parameters, including short-chain fatty acid (SCFA) production, acidification trends, and proteolytic activity, were measured using a high-resolution metabolomic profiling via the Laser-Assisted Rapid Evaporative Ionization Mass Spectrometry (LA-REIMS).

**Results:**

Descriptive observations revealed distinct and complementary metabolic patterns. Microbiotal supplementation enhanced acidification in both the proximal and distal colon, with stimulation of acetate production in the proximal colon. Treatment with *L. reuteri* stimulated lactate production while reducing acetate and propionate levels. The synbiotic treatment showed combined effects, increasing acetate in the distal colon and producing metabolic shifts, as determined by LA-REIMS analysis. All treatments showed trends toward increased proteolytic markers in the distal colon.

**Conclusion:**

This pilot *in vitro* feasibility study demonstrates that prebiotics, probiotics, and synbiotics produce distinct metabolic fingerprints *in vitro,* warranting future validation through studies with multiple donors, independent SCIME™ runs, and *in vivo* trials to assess generalizability and explore potential applications in canine gastrointestinal research.

## 1 Introduction

The gut microbiota (1), a vital and intricate microbial ecosystem, is essential for canine health. It carries out a multitude of physiological functions, including the fermentation of dietary substrates, production of SCFAs, synthesis of vitamins, immune modulation, and protection against pathogenic microbes. The imbalance of this microbiota, known as dysbiosis, is linked to various gastrointestinal and systemic diseases, such as inflammatory bowel disease (IBD), small intestinal bacterial overgrowth (SIBO), chronic diarrhea, and obesity (2–7). Dysbiosis can be generally defined as an alteration in gut microbiota composition (8, 9). It can be categorized as transient or pathological, with the latter associated with intestinal diseases and characterized by disruptions in mucosal bacteria (9).

Prebiotics, such as non-digestible oligosaccharides (NDOs), have emerged as a promising strategy to modulate the gut microbiota. These substances have the unique ability to selectively stimulate the growth and activity of beneficial microbes, thereby enhancing saccharolytic fermentation and SCFA production (10). However, the current prebiotics are predominantly fermented in the proximal colon, where the metabolism is mostly saccharolytic (11), with only limited amounts reaching the distal colon region. Given that most chronic colonic diseases (for instance, ulcerative colitis and colorectal cancer) originate in the distal colon, there is a growing interest in discovering prebiotics that exert biological activity throughout the entire colon, such as specific prebiotics including some inulins and resistant starches (12, 13) that resist digestion in the small intestine and are fermented in the distal colon (14).

Probiotics are recognized for their role in improving human health, most likely by transiently modifying the gut microbiota composition. Indeed, they are defined as live microorganisms that confer health benefits when consumed in adequate amounts, modulate microbial populations, and reduce harmful fermentation byproducts (15).

Synbiotics, a combination of prebiotics and probiotics, leverage the synergistic effects of both components to maximize therapeutic outcomes. The potential of synbiotics to enhance the production of health-related bacterial metabolites and evaluate prebiotic and probiotic effects in the gut is a reason for optimism about the future of canine health. In May 2019, the International Scientific Association for Probiotics and Prebiotics (ISAPP) updated the definition of synbiotics to “a mixture comprising live microorganisms and substrate(s) selectively utilized by host microorganisms that confers a health benefit on the host.” ISAPP emphasizes that defining synbiotics as simply a mixture of probiotics and prebiotics could limit the development of products aimed at improving human and animal health (16).

The metabolome is a compilation of all primary and secondary metabolites, which are regarded as the final recipients of genetic information, thereby also overarching information about the transcriptome and proteome. Notably, the metabolome is not predefined but rich and dynamic in response to many extrinsic factors such as the microbial community, physical activity, dietary pattern, and the environment (17). The metabolome is quite complex, with thousands of metabolites at very different concentration levels and with huge physicochemical diversity (18). Moreover, these metabolome constituents have been assigned a significant role in many biological processes. As such, metabolomics is concerned with the comprehensive analysis of metabolites, providing a direct functional read-out of the physiological status of an organism and, in principle, ideally suited to describe someone’s health status, from normal physiology to diverse pathophysiologies (19). In this context, with the canine microbiological community being an important extrinsic factor, insights into modulating the microbiome’s metabolic activities are valuable to support a metabolically healthy condition.

The primary objective of this pilot feasibility study was to assess the technical applicability of the SCIME™ platform combined with LA-REIMS metabolomics for investigating the metabolic responses of canine gut microbiota to three dietary supplements. These products include Microbiotal, product M, a formulation containing prebiotics (e.g., oligofructose, inulin) and a postbiotic (thermally inactivated *Lactobacillus reuteri*) manufactured by NBF Lanes, Milan, Italy, (20), a probiotic, product P, (*L. reuteri,* NBF Lanes, Milan, Italy) (21, 22), and a synbiotic, product M + P, (Microbiotal + *L. reuteri*). The selection of *L. reuteri* was based on its documented safety and efficacy in dogs, as previously reported (21, 22). Similarly, the Microbiotal formulation has been shown to modulate the intestinal microbiota in sled dogs (20).

Experiments were carried out using the SCIME™ technology platform, an innovative *in vitro* colonic fermentation model. This model utilizes fecal inoculum from healthy canine donors, enabling preliminary investigation of how the products may affect gut microbiota. The SCIME™ platform was selected for its ability to replicate the regional conditions of the canine colon in a controlled setting, facilitating the study of microbial metabolism without the ethical and logistical challenges posed by *in vivo* trials. While the model does not fully capture host-derived factors, such as immune responses, and the current study design employs a single donor and single SCIME™ run per treatment condition, it offers high reproducibility for technical validation and provides valuable preliminary insights into microbial fermentation processes (23–27).

This study continues the previous work by Gramenzi et al. (23) which analyzed the same test products, both alone and in combination, for their ability to modulate the microbial community of the canine gut microbiota. The hypothesis guiding this feasibility investigation posits that Microbiotal product M, probiotic product P, and synbiotic supplements (specifically the M + P product) may produce distinct metabolic fingerprints in the canine gut microbiota *in vitro*, depending on the region of the colon (proximal versus distal), as a foundation for future *in vivo* validation studies.

## 2 Materials and methods

### 2.1 Donor selection and fecal sample collection

A healthy dog with an optimal body condition score (BCS) weighing 20.4 kg was selected as the fecal donor. The donor was a 7-year-old neutered male mixed-breed dog (a privately owned dog that was temporarily loaned for this research) presenting a BCS of 4.5 according to the official 9-point scale (1–9) established by the World Small Animal Veterinary Association (WSAVA) Global Nutrition Guidelines^1^. The donor was considered clinically healthy based on the absence of gastrointestinal disorders in its medical history, no antibiotic treatment within the previous 6 months, no history of diarrheal episodes, and no use of dietary supplements. No baseline microbiota analysis was performed to verify the healthy status of the donor’s gut microbiota composition. Prior to fecal sample collection, the donor was examined by an expert nutritionist and subjected to a personalized and balanced diet for 2 weeks to maintain the appropriate BCS and standardize gut microbiota composition. The daily maintenance energy requirement of the donor dog was calculated at 998 kcal using Myvetdiet software, and the animal was fed 290 g per day of a commercial dry diet (Royal Canin Sterilised Adult Medium Dog), providing all essential macro- and micronutrients required for the specific physiological condition of the donor. Fresh fecal samples were collected nearly immediately after defecation to ensure viability of the microbes in a single collection event (26), homogenized with phosphate-buffered saline (PBS, 1:10 w/v), and supplemented with 0.05% cysteine-HCl to minimize oxidative stress. Samples were stored at 4 °C and processed within 6 h to preserve microbial viability.

### 2.2 Study framework

For this investigation, the Simulator of Canine Intestinal Microbial Ecosystem (SCIME™) was employed as a laboratory model to replicate the distinct regions of the canine gastrointestinal tract (GIT). This sophisticated system is structured into interconnected compartments corresponding to the stomach, small intestine, proximal colon, and distal colon, enabling a detailed examination of microbial dynamics (23).

### 2.3 Adaptation of the SCIME™ platform

The SCIME™ model, derived from the established Simulator of the Human Intestinal Microbial Ecosystem (SHIME®), was specifically modified to mimic the physiological environment of the canine GIT (28–30).

Key customizations included:

1. Temperature Regulation: The system was maintained at a stable temperature of 39 °C, reflecting the natural body temperature of dogs.
2. pH and Retention Time Adjustments: Each compartment’s conditions were tailored to align with the canine digestive process. For instance, the proximal colon was maintained at a pH range of 5.7–5.9, while the distal colon’s pH was set between 6.6 and 6.9. The retention times were set to simulate canine physiology: 12 h for the stomach/small intestine, 24 h for the proximal colon, and 30 h for the distal colon (26).
3. Microbial Inoculation: Fresh fecal microbiota obtained from the healthy adult canine donor was used as the inoculum. This setup facilitated a dynamic simulation of microbial communities under controlled experimental conditions.

The model also incorporated both luminal and mucosal compartments, recognizing the significance of mucosa-associated microbiota in gut barrier functionality and immune system interactions (26, 31, 32). An enhanced configuration, termed the Triple-M-SCIME™ (24–26), allowed simultaneous testing of three distinct experimental conditions. Each segment comprised a succession of reactors simulating the stomach and small intestine combined (St + Si), the proximal colon (PC), and the distal colon (DC) as already described by Gramenzi et al. in 2024 (23).

### 2.4 Experimental workflow

The study was conducted over three phases:

1. Stabilization Period (3 Weeks): All three units were inoculated with the same canine fecal sample. This period allowed the microbial communities to adapt within each unit’s reactors, achieving stable populations in both colonic compartments.
2. Control Period (2 Weeks): All three units received the standard nutrient matrix without supplementation. Baseline data were collected to establish microbial composition and metabolic activity. During this control period, samples were collected at regular intervals as follows: sampling occurred three times per week (on days 1, 3, and 5 of each week), resulting in a total of six measurements over the 2-week control period (six technical replicates per treatment unit).
3. Treatment Period (2 Weeks): Three dietary interventions were applied, one per unit:
  - Microbiotal (product M) (20): Administered as one tablet daily (1,250 mg per tablet), containing: fructooligosaccharides (FOS) 250 mg, microencapsulated tributyrate 60 mg, inulin 180 mg, polyphenols from red orange 120 mg, thermally inactivated *Lactobacillus reuteri* NBF1 (tyndallized) 100 mg, vitamin B12 15 μg, and folic acid 0.3 mg per tablet (21).
  - Probiotic (*L. reuteri*, product P) (21, 22): Delivered at a dose corresponding to one capsule daily (375 mg per capsule), containing: *Lactobacillus reuteri* DSM 32203 at 1.25 × 10^10^ CFU; *Saccharomyces cerevisiae* MUCL 39885 at 7.5 × 10^8^ CFU; *Bacillus velezensis* DSM 15544 at 2.5 × 10^8^ CFU; *Enterococcus faecium* DSM10663-NCIMB 10415 at 7 × 10^7^ CFU; *Lactobacillus reuteri* NBF1 thermally inactivated (tyndallized) 10 mg; and Vitamin B12 5 μg per capsule (23).
  - Combination Treatment, product M + P: A synbiotic approach combining product M and probiotic product P formulations.

During the treatment period, samples were collected following the same protocol as the control period: three times per week (on days 1, 3, and 5 of each week), resulting in six technical replicates over the 2-week treatment period per treatment unit.

The Triple-M-SCIME™ system allowed for the simultaneous testing of three conditions in parallel. Throughout the treatment phase, the SCIME™ nutrient medium was supplemented with the respective dietary interventions. The dosing regimen in this *in vitro* model involved daily supplementation directly into the nutrient medium.

### 2.5 Measured parameters

- pH Monitoring: pH was recorded at 0, 24, 48, 72 h, and weekly to evaluate acidification trends.
- SCFA Quantification: Gas chromatography–mass spectrometry (GC–MS) quantified acetate, propionate, and butyrate concentrations. Lactate levels were also measured to assess shifts in fermentation pathways.
- Proteolytic Byproducts: Ammonium and branched SCFAs (isobutyrate, isovalerate) were analyzed as markers of proteolytic activity.
- Metabolomic Profiling: LA-REIMS (Laser Ablation-Rapid Evaporative Ionization Mass Spectrometry) provided high-resolution metabolic fingerprints, identifying saccharolytic and proteolytic fermentation markers, as well as metabolic shifts specific to treatments.

### 2.6 Description of statistics

Data are presented as mean ± standard deviation of measurements taken from each SCIME™ unit over the respective period (*n* = 6 technical replicates per period). Descriptive comparisons between control and treatment periods were performed. *p*-values < 0.05 are reported descriptively to indicate apparent differences observed within this experimental run.

### 2.7 LA-REIMS methodology and working principle

Individual *in vitro* fluid samples (*n* = 72) were thawed at 4 °C and shortly vortexed. Per sample, 200 μL was transferred into a well of a 96-well plate and subjected to LA-REIMS analysis. The LA-REIMS platform used a MID infrared laser system (Opolette™ HE2940, OPOTEK) that consisted of a Q-switched Nd: YAG laser pumping Optical Parametric Oscillator (OPO). The laser energy was transmitted into the sample through free space optics, including a series of metallic-coated mirrors and a Plano-convex lens. Hereby, the laser ablation process is initiated based on the laser-emitted infrared wavelength regime that excites the most intense vibrational band (oxygen-hydrogen stretching mode) of the water molecules contained by the sample under investigation. This induces so-called matrix-assisted desorption and ionization of intact biomolecules. Subsequently, the aerosol produced is transferred to the REIMS platform. Mass analysis was conducted using a Xevo G2-XS Quadrupole Time-of-Flight (QToF) mass spectrometer (Waters Corporation), operated in negative ionization mode, and applying an m/z scan range from 50 to 1,200 Da. For sampling, an automated, versatile 96-multi-well plate-based platform was incorporated in the standard LA-REIMS workflow (33).

**Figure 1:**
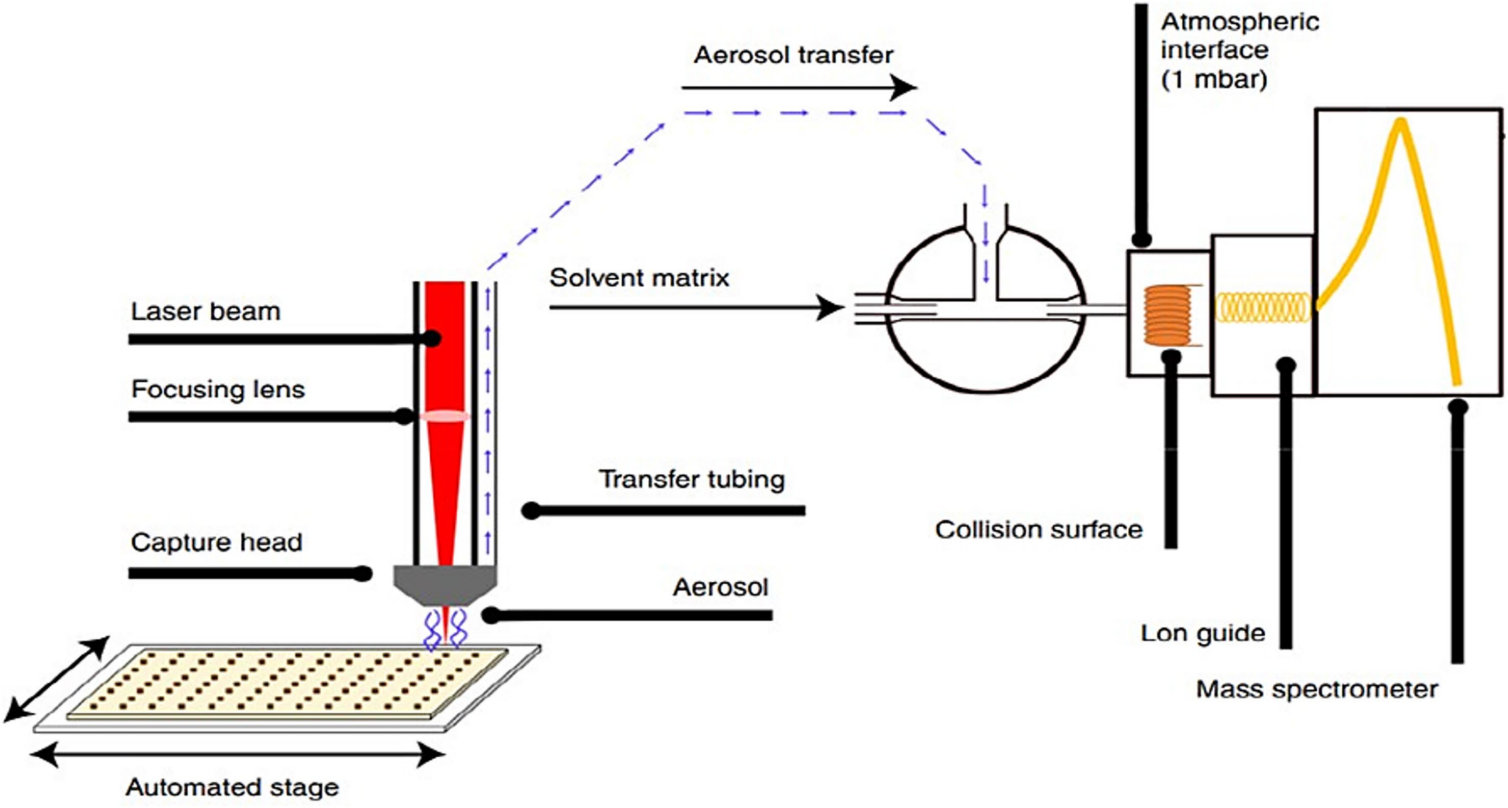
Technical scheme of the LA-REIMS setup whereby a laser beam is focused onto a sample and is placed on an automated stage equipped to handle 96-well plates. Rapid heating and evaporation occur, and the resulting analyte-containing aerosol is aspirated toward the mass spectrometer under the instrument’s internal vacuum. The vapor is mixed with a solvent matrix containing a lock mass/external standard and collides with a heated collision surface of the REI source to form gas-phase ions for analysis. These ions are analyzed using a QToF-MS instrument (23).

The typical setup of the LA-REIMS platform and the working principle are presented in Figure 1.

### 2.8 Quality assurance

The REIMS instrument was calibrated in sensitivity mode prior to analysis according to the manufacturer’s standard instructions (Waters Corporation, UK). However, biological samples were analyzed in a randomized order organized per colon region (proximal and distal colon).

A pool of *in vitro* fluid was assembled by taking 200 μL from every biological sample. This pool was thoroughly vortexed and used as a quality control sample (QC). Twenty runs of the QC sample were included to condition the LA-REIMS instrument (i.e., external QC; eQC), whereas two runs were included after every (max.) ten biological samples to monitor instrument performance (i.e., internal QC; iQC).

An external standard, i.e., leucine-enkephalin (0.25 ng μL-1), was used during REIMS analysis, offering opportunities for lock mass correction and correction of signal intensity drift.

### 2.9 LA-REIMS data processing and multivariate statistics

LA-REIMS data (.raw files) were first processed using MassLynx® V4.1 Progenesis® Bridge (Waters Corporation, UK), whereby extracted ion chromatograms were created, and background subtraction was performed. To this end, a threshold of 5 e (34) was generally set to achieve ion chromatogram extraction (i.e., selection of burn-only signals). Background subtraction was performed to remove the signals associated with the ‘noise’ (35).

Following ion chromatogram extraction, ‘bridged’ data (.raw files) were processed by Progenesis® QI V2.3 (Waters Corporation, UK) to list the detected m/z features and their relative abundances across samples. To this end, the automatic sensitivity mode for peak picking (default value) was applied. The m/z features refer to individually detected signals, which may correspond to a particular ionization adduct, isotope, in-source fragment, etc. As such, various features may relate to a single metabolite. The abundances of the detected m/z features were normalized based on all ion compounds. In that way, the signals underlying a specific burn were normalized against the ‘success’ of the laser ablation process and associated overall detectability of metabolites.

Normalized data were subjected to multivariate statistical analysis using SIMCA 17 (Sartorius, Germany). Here, data were pre-processed, thereby performing log transformation to induce normal distributions and unit variance scaling (1/SD, with SD being the standard deviation) to standardize the range of signal intensities. Unsupervised Principal Component Analysis (PCA-X) was executed to assess the natural patterning of samples and reveal potential outliers (based on Hotelling’s T2 criterion). Orthogonal Partial Least Squares Discriminant Analysis (OPLS-DA) was used to differentiate samples according to experimental conditions in a supervised fashion. The validity of the OPLS-DA models was verified by permutation testing (*n* = 100), cross-validated analysis of variance (*p*-value < 0.05), and the quality parameter Q^2^(Y) (≥0.5). In general, the model performance is described by R^2^(X) (the predictive and orthogonal variation in X-values, i.e., m/z features), R^2^(Y) (the ability of the model to predict the Y-data for the specifically used dataset, i.e., predicting the sample classification) and Q2 (the ability of the model to correctly predict the Y-data if an external dataset would be considered) (36).

## 3 Results

### 3.1 pH modulation

**Figure 2:**
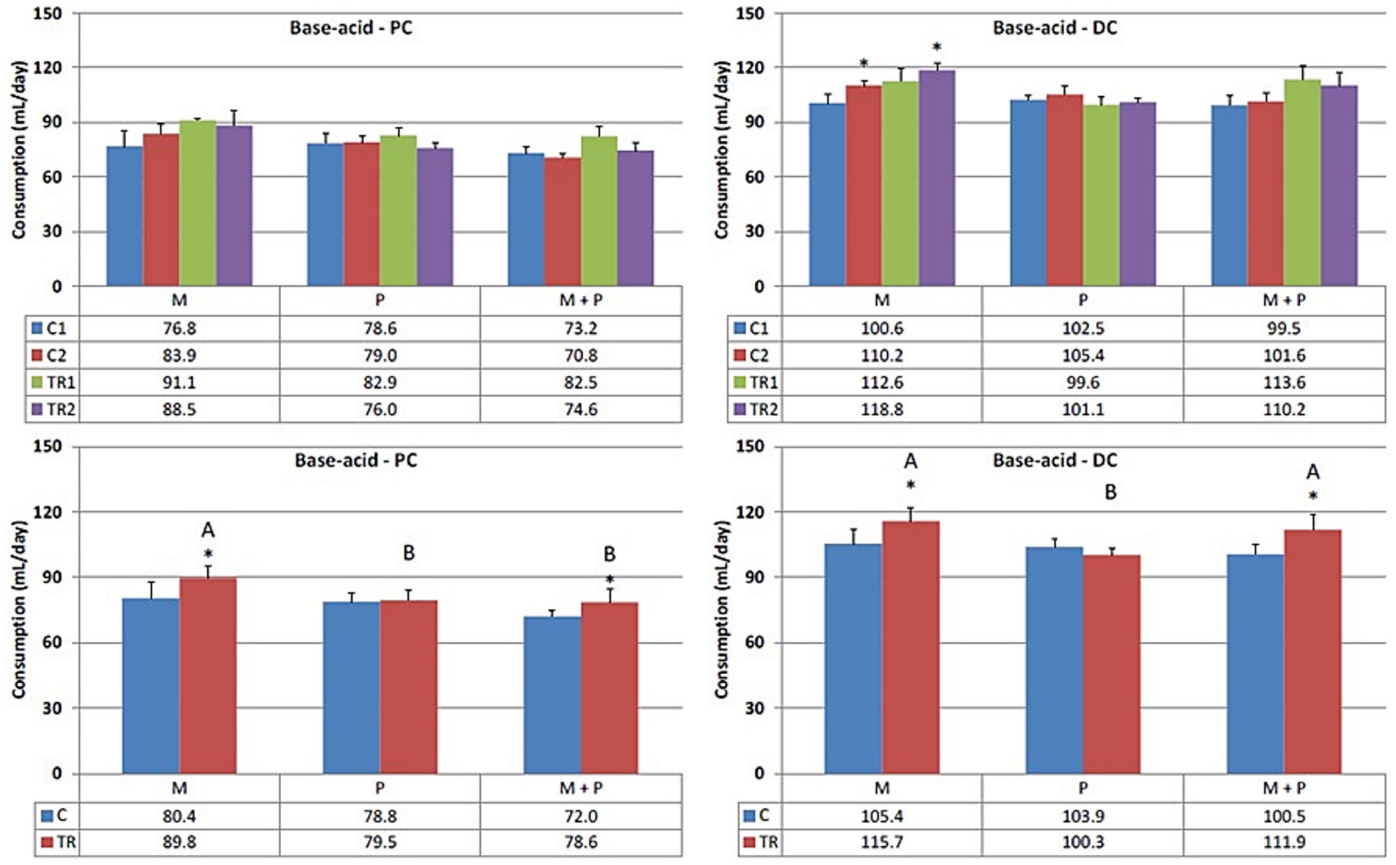
Effect of treatment with the different test products (Microbiotal, M; probiotic, P; and their combination, M + P) on base–acid consumption (mL/day) in the proximal (PC; **left**) and distal colon (DC; **right**). **Top**: average weekly base–acid consumption during control (C1-C2) and treatment (TR1-TR2) weeks (*n* = 3). * Indicates apparent differences observed within this SCIME™ run; descriptive *p* < 0.05. **Bottom**: average base–acid consumption over the entire control (C; *n* = 6 technical replicates) and treatment (TR; *n* = 6 technical replicates) period. * Indicates apparent differences observed within this SCIME™ run, while different letters indicate descriptive differences between treatments; *p* < 0.05 reflects technical variability only.

In both the proximal and distal colon (Figure 2), supplementation of the Microbiotal test product, M, showed increased acidification, irrespective of co-supplementation with the probiotic test compound, M + P. Supplementation of the probiotic test product P did not notably alter base consumption in either colon region. It is important to note that the pH in the proximal colon was consistently at the upper limit (pH 5.9) during the control period, due to lower levels of fermentable substrates. During the treatment period, the addition of fermentable substrates acidified the proximal colon content to the lower limit (pH 5.7). Consequently, the transfer of this more acidic suspension to the distal colon necessitated greater base consumption to maintain the preset pH interval.

### 3.2 SCFA profiles

**Figure 3:**
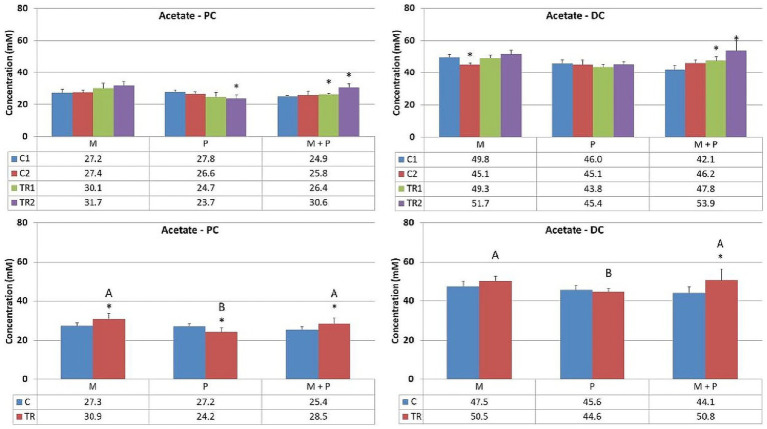
Effect of treatment with the different test products (Microbiotal, M; probiotic, P; and their combination, M + P) on acetate production (mM) in the proximal (PC; **left**) and distal colon (DC; **right**). **Top**: average weekly acetate production during control (C1-C2) and treatment (TR1-TR2) weeks (*n* = 3). * Indicates apparent differences observed within this SCIME™ run; descriptive *p* < 0.05. **Bottom**: average acetate production over the entire control (C; *n* = 6 technical replicates) and treatment (TR; *n* = 6 technical replicates) period. * Indicates apparent differences observed within this SCIME™ run, while different letters indicate descriptive differences between treatments; *p* < 0.05 reflects technical variability only.

**Figure 4:**
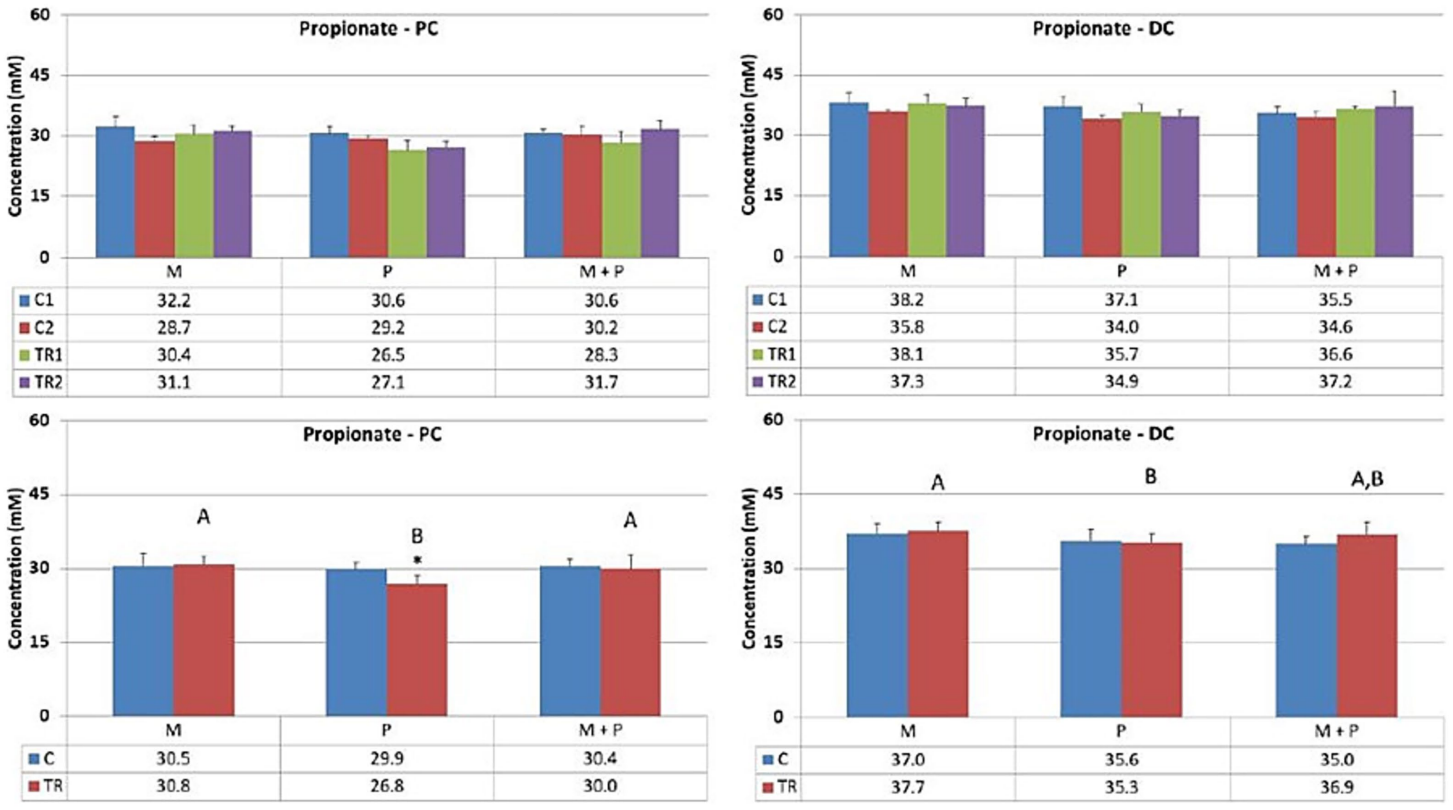
Effect of treatment with the different test products (Microbiotal, M; probiotic, P; and their combination, M + P) on propionate production (mM) in the proximal (PC; **left**) and distal colon (DC; **right**). **Top**: average weekly propionate production during control (C1-C2) and treatment (TR1-TR2) weeks (*n* = 3). * Indicates apparent differences observed within this SCIME™ run; descriptive *p* < 0.05. **Bottom**: average propionate production over the entire control (C; *n* = 6 technical replicates) and treatment (TR; *n* = 6 technical replicates) period. * Indicates apparent differences observed within this SCIME™ run, while different letters indicate descriptive differences between treatments; *p* < 0.05 reflects technical variability only.

**Figure 5:**
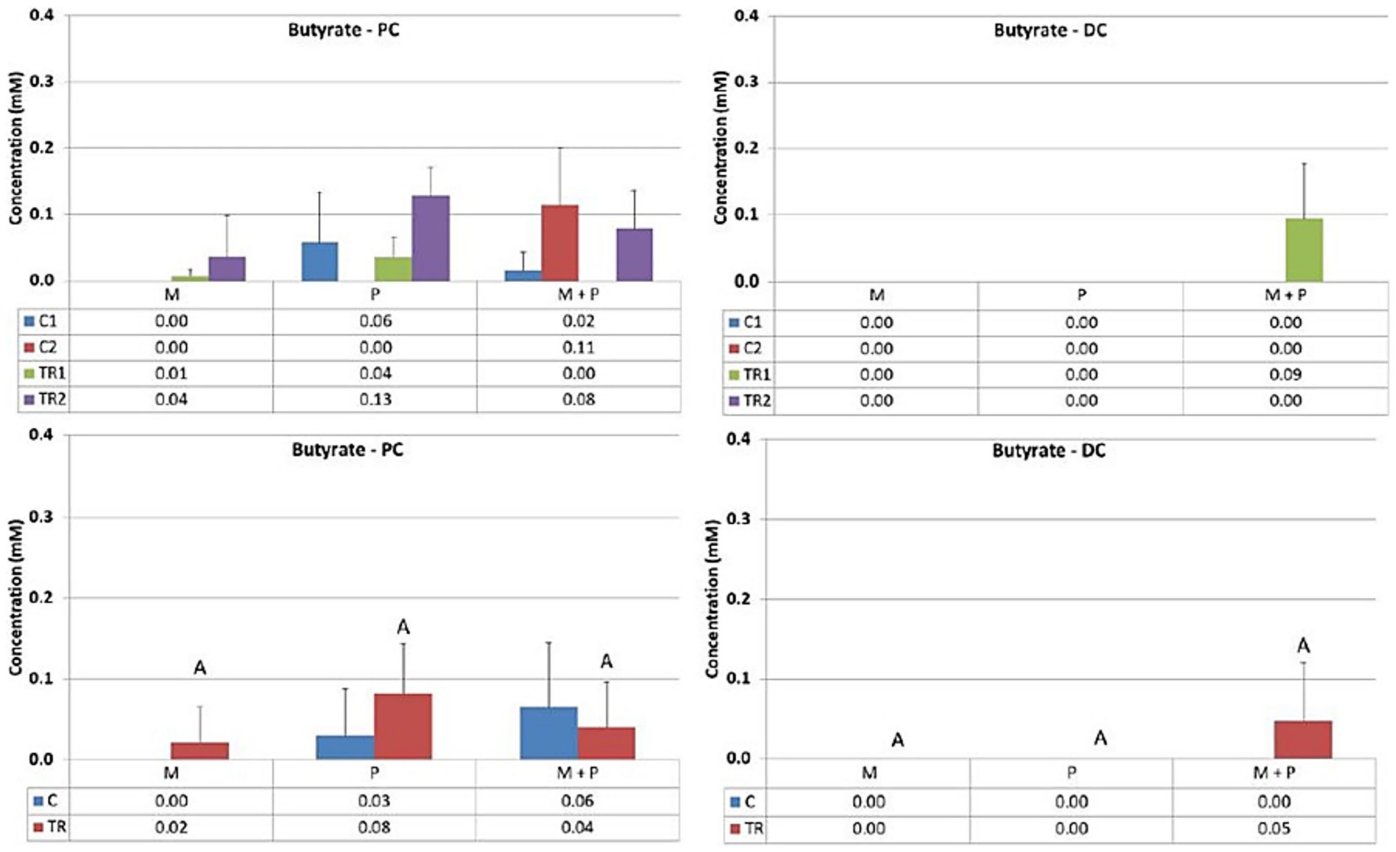
Effect of treatment with the different test products (Microbiotal, M; probiotic, P; and their combination, M + P) on butyrate production (mM) in the proximal (PC; **left**) and distal colon (DC; **right**). **Top**: average weekly butyrate production during control (C1-C2) and treatment (TR1-TR2) weeks (*n* = 3). * Indicates apparent differences observed within this SCIME™ run; descriptive *p* < 0.05. **Bottom**: average butyrate production over the entire control (C; *n* = 6 technical replicates) and treatment (TR; *n* = 6 technical replicates) period. * Indicates apparent differences observed within this SCIME™ run, while different letters indicate descriptive differences between treatments; *p* < 0.05 reflects technical variability only.

**Figure 6:**
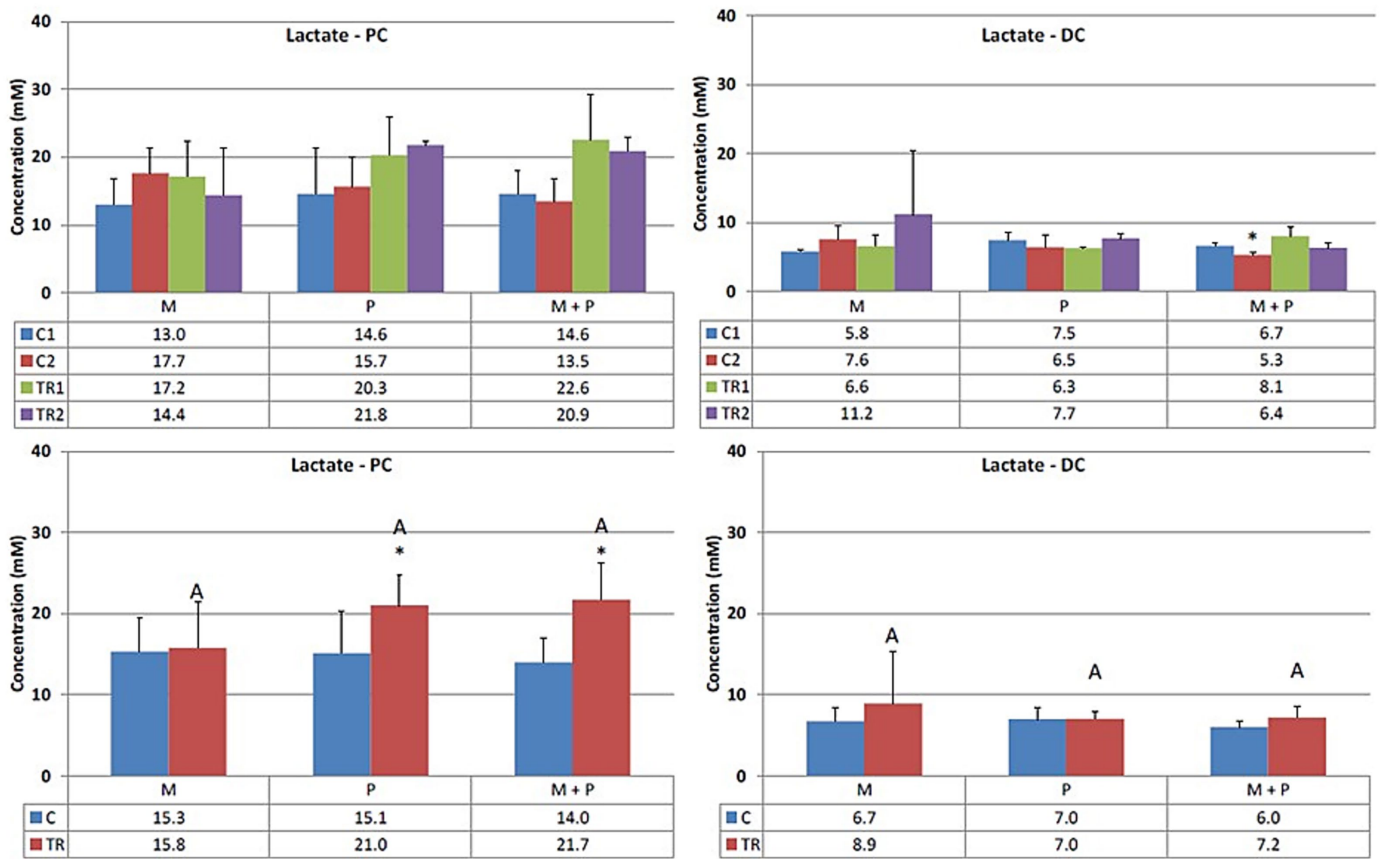
Effect of treatment with the different test products (Microbiotal, M; probiotic, P; and their combination, M + P) on lactate production (mM) in the proximal (PC; **left**) and distal colon (DC; **right**). **Top**: average weekly lactate production during control (C1-C2) and treatment (TR1-TR2) weeks (*n* = 3). * Indicates apparent differences observed within this SCIME™ run; descriptive *p* < 0.05. **Bottom**: average lactate production over the entire control (C; *n* = 6 technical replicates) and treatment (TR; *n* = 6 technical replicates) period. * Indicates apparent differences observed within this SCIME™ run, while different letters indicate descriptive differences between treatments; *p* < 0.05 reflects technical variability only.

Descriptive patterns in SCFA production were observed:

- Acetate (Figure 3) is primarily one of the key metabolites formed during primary substrate fermentation. Microbiotal test product, M, showed increased acetate production in the proximal colon. In the distal colon, albeit a numerical increase in acetate was observed with Microbiotal treatment M alone, the most pronounced increase was achieved upon co-supplementation with the probiotic test product P; descriptive *p* < 0.05. On the other hand, the probiotic, P, showed a decrease in acetate production in the proximal colon. Since the supplemented *L. reuteri* P typically produces lactate, this could indicate a shift in the primary degradation patterns within this *in vitro* system.
- Propionate (Figure 4) can be produced by a wide range of gut microbes. Propionate production decreased upon supplementation of the *L. reuteri* test product, P, in the proximal colon (descriptive *p* < 0.05), potentially indicating altered cross-feeding interactions in the microbial community within this *in vitro* model. Co-supplementation of the Microbiotal test product M (i.e., M + P) appeared to moderate this effect. Upon supplementation of Microbiotal, M, propionate levels remained relatively stable. The test products had minimal apparent effects on propionate levels in the distal colon.
- Butyrate (Figure 5) is produced by Clostridium clusters IV and XIVa members. Butyrate levels remained around or just below the detection limit. The different test products did not impact butyrate levels in the proximal or distal colon within this *in vitro* system.
- Lactate (Figure 6) upon supplementation of the Microbiotal test product, M, lactate levels remained largely unchanged in both the proximal and distal colon. Supplementation of the *L. reuteri* test product, P, irrespective of Microbiotal M co-supplementation (i.e., M + P), showed increased lactate levels in the proximal colon (descriptive *p* < 0.05), indicating that the probiotic, P, influenced the fermentation patterns of the microbial community. Since lactate levels in the distal colon remained largely unaffected, the probiotic, P, mainly impacted the proximal colon within this *in vitro* system.

### 3.3 Proteolytic fermentation

Both the production of ammonium and branched SCFA (the sum of isobutyrate, isovalerate, and isocaproate) result from protein degradation and reflect the proteolytic activity of the gut microbiota.

**Figure 7:**
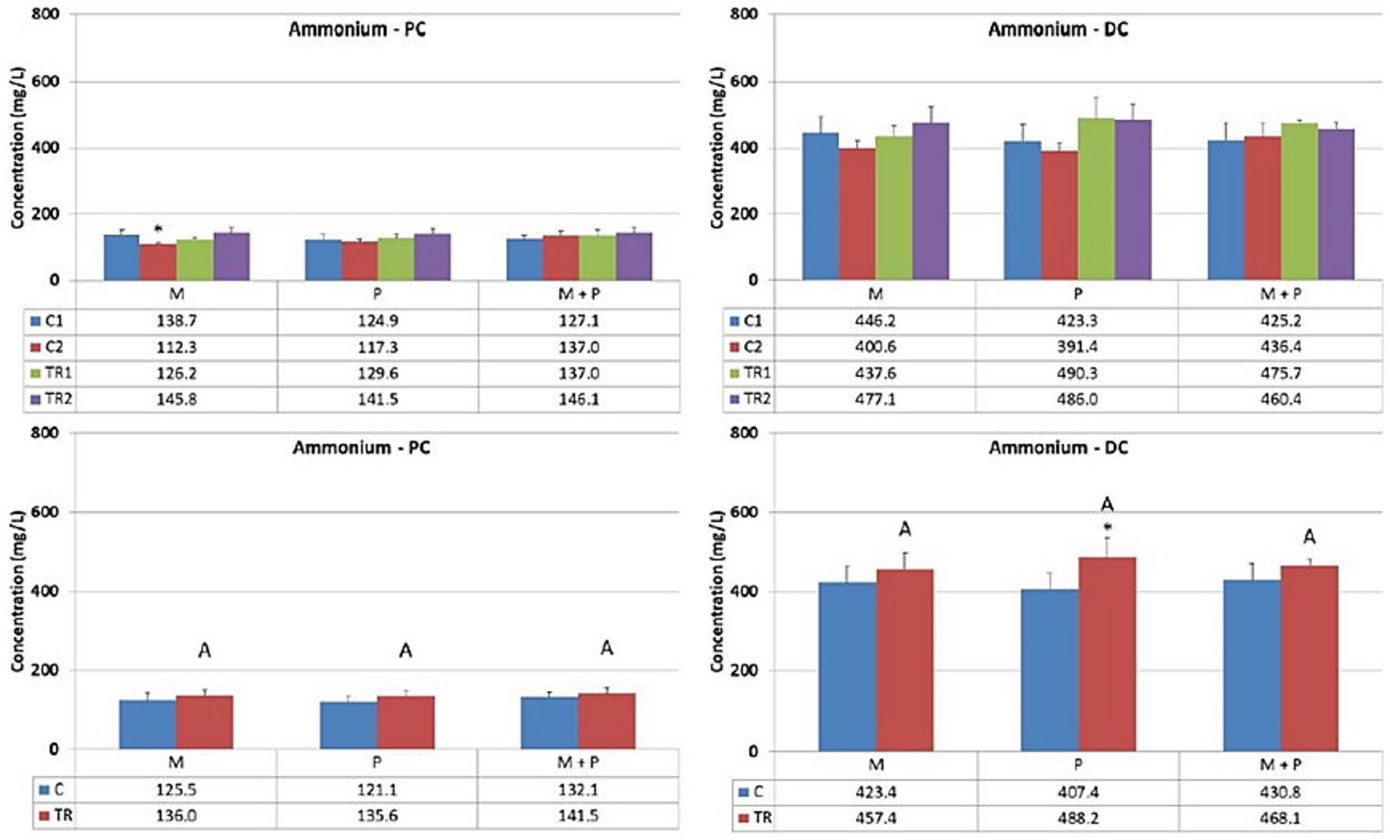
Effect of treatment with the different test products (Microbiotal, M; probiotic, P; and their combination, M + P) on ammonium production (mg/L) in the proximal (PC; **left**) and distal colon (DC; **right**). **Top**: average weekly ammonium production during control (C1-C2) and treatment (TR1-TR2) weeks (*n* = 3). * Indicates apparent differences observed within this SCIME™ run; descriptive *p* < 0.05. **Bottom**: average ammonium production over the entire control (C; *n* = 6 technical replicates) and treatment (TR; *n* = 6 technical replicates) period. * Indicates apparent differences observed within this SCIME™ run, while different letters indicate descriptive differences between treatments; *p* < 0.05 reflects technical variability only.

**Figure 8:**
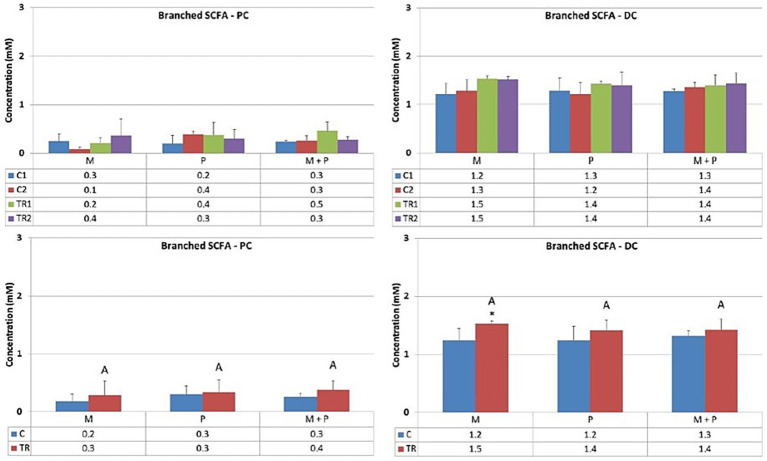
Effect of treatment with the different test products (Microbiotal, M; probiotic, P; and their combination, M + P) on branched SCFA production (mM) in the proximal (PC; **left**) and distal colon (DC; **right**). **Top**: average weekly branched SCFA production during control (C1-C2) and treatment (TR1-TR2) weeks (*n* = 3). * Indicates apparent differences observed within this SCIME™ run; descriptive p < 0.05. **Bottom**: average branched SCFA production over the entire control (C; *n* = 6 technical replicates) and treatment (TR; *n* = 6 technical replicates) period. * Indicates apparent differences observed within this SCIME™ run, while different letters indicate descriptive differences between treatments; *p* < 0.05 reflects technical variability only.

Concerning ammonium levels (Figure 7), ammonium concentrations remained stable in the proximal colon upon treatment with the different test products. These observations were consistent with the effects on branched SCFA production (Figure 8). In the distal colon, a trend toward increased proteolytic fermentation was observed when supplemented with any test product, with apparent differences noted in the probiotic, P, arm (ammonium levels, descriptive *p* < 0.05) and the Microbiotal, M, arm (branched SCFA levels, descriptive *p* < 0.05) within this *in vitro* system.

### 3.4 Metabolomic shifts

#### 3.4.1 LA-REIMS analysis revealed treatment-specific metabolic fingerprints and significant regional differences

**Figure 9:**
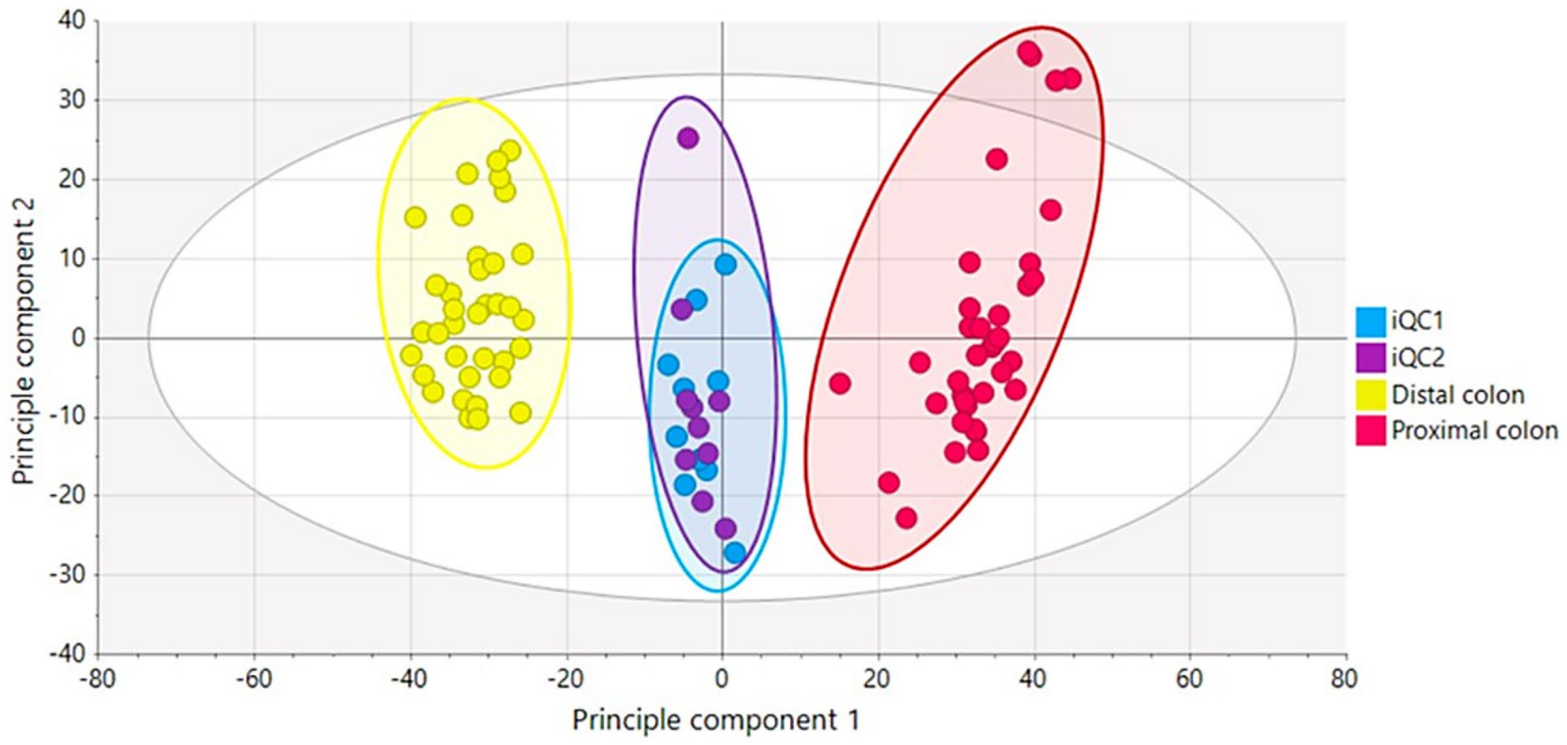
PCA-X score plot based on the LA-REIMS data (negative ionization mode) for the biological samples (*n* = 72) and iQC-samples (*n* = 20). The plot shows a clear separation between samples from the proximal colon (red), distal colon (yellow), and internal quality control samples (iQC 1 and 2). Principal Component 1 (PC1, 53.9%) and Principal Component 2 (PC2, 11.0%) together explain 64.9% of the total variance in the dataset. One outlier sample was identified but retained for analysis.

High-resolution metabolic profiling via LA-REIMS generated comprehensive fingerprints of the luminal and mucosal microbiota, comprising 1,603 distinct metabolic features (represented by unique m/z signals). Based on these fingerprints, multivariate statistics were performed (37, 38), and unsupervised PCA-X modeling was used to reveal the natural patterning of samples. The PCA-X score plot (Figure 9) revealed a clear and primary metabolic segregation of samples according to the simulated colon region, with Principal Component 1 (PC1) explaining 53.9% of the total variance. This stark metabolic divergence between the proximal and distal colon was also statistically confirmed when a validated supervised OPLS-DA model was performed, underscoring the fundamental influence of the colonic environment on microbial metabolism. Given this pronounced regional variation, all subsequent analyses of treatment effects were stratified by colon region, and a more detailed investigation of these effects was conducted through supervised OPLS-DA modeling, which compared each treatment group against its respective control across all time points. The results of these pairwise comparisons are summarized in Tables 1, 2.

**Table 1:** Description of the OPLS-DA models comparing LA-REIMS metabolic fingerprints from the proximal colon following treatment with the different test products and their associated controls.

Model (tp + t0)	R^2^X	R^2^Y	Q2Y	*p*-value	Permutation testing	Conclusion
Control 1 vs. Treatment 1 (0 + 0)	No model could be generated					Not valid
Control 2 vs. Treatment 2 (1 + 0)	0.418	0.694	0.560	0.024	Valid	Valid
Control 3 vs. Treatment 3 (1 + 2)	0.602	0.989	0.815	0.033	Valid	Valid

tp, predictive principal component; to, orthogonal principal component. Treatment 1: Microbiotal test product M; Treatment 2: *L. reuteri* probiotic test product P; Treatment 3: Microbiotal test product M + *L. reuteri* probiotic P, namely M + P synbiotic treatment.

**Table 2:** Description of the OPLS-DA models comparing LA-REIMS metabolic fingerprints from the distal colon following treatment with the different test products and their associated controls.

Model (t_p_ + t_0_)	R^2^X	R^2^Y	Q^2^Y	*p*-value	Permutation testing	Conclusion
Control 1 vs. Treatment 1 (1 + 1)	0.484	0.913	0.619	0.107	Valid	Not valid
Control 2 vs. Treatment 2 (0 + 0)	No model could be generated					Not valid
Control 2 vs. Treatment 3 (1 + 2)	0.0611	0.992	0.801	0.040	Valid	Valid

tp, predictive principal component; to, orthogonal principal component. Treatment 1: Microbiotal test product M; Treatment 2: *L. reuteri* probiotic test product P; Treatment 3: Microbiotal test product M + *L. reuteri* probiotic P, namely M + P synbiotic treatment.

Overall, LA-REIMS-based analysis revealed distinct metabolic patterns between the proximal and distal colon regions within this *in vitro* system. Supervised multivariate modeling (OPLS-DA) indicated that in the proximal colon, distinct metabolic fingerprints compared to control were observed for the treatment with pure *L. reuteri,* product P, and for its co-supplementation with the Microbiotal, product M, (i.e., synbiotic product M + P). In contrast, treatment with the Microbiotal product M alone did not generate a validated OPLS-DA model in this region. A distinct metabolic fingerprint with validated OPLS-DA differentiation was revealed in the distal colon exclusively upon treatment with *L*. *reuteri* probiotic P in co-supplementation with the Microbiotal test product M, namely the symbiotic product M + P. The other treatment conditions did not produce validated OPLS-DA models in the distal colon within this single SCIME™ run.

## 4 Discussion

This pilot feasibility study explores the technical application of the SCIME™ platform combined with LA-REIMS metabolomics to investigate metabolic responses of canine gut microbiota to dietary supplements. The study examined the effects of prebiotics included in product M (along with a postbiotic), the probiotic P, and the synbiotic product M + P using a novel *in vitro* model, the Simulator of Canine Intestinal Microbial Ecosystem (SCIME™) (23–27). The findings provide preliminary technical insights into the potential roles of these dietary interventions in modulating microbial metabolic activity *in vitro*, with implications for future *in vivo* validation studies in canine gastrointestinal health management.

The results demonstrate that supplementation with the Microbiotal product M significantly enhances acidification in both the proximal and distal colonic compartments within this *in vitro* system. This acidification correlates with an increase in acetate production, particularly in the proximal colon. Acetate, a short-chain fatty acid (SCFA), is a critical end product of saccharolytic fermentation and plays a pivotal role in maintaining intestinal homeostasis and providing an energy source for colonocytes and a potential substrate for lipid synthesis (23, 39). These findings align with existing literature emphasizing the benefits of prebiotics in promoting saccharolytic fermentation and SCFA production (10). This pilot study provides preliminary insights into how Microbiotal (product M), despite containing several components that are difficult to isolate, may influence metabolic activity throughout the colon *in vitro*. This aspect is particularly important given that many chronic colonic diseases originate in the distal colon (40). The observed increase in acidification and SCFA production *in vitro* suggest that specific prebiotics warrants further investigation through *in vivo* studies as a potential preventative strategy against dysbiosis-associated disorders, such as irritable bowel syndrome (IBS).

The *L. reuteri* probiotic P elicited distinct changes in fermentation dynamics in this *in vitro* model, particularly increasing lactate production in the proximal colon. Lactate, often regarded as a transitional metabolite, serves as a substrate for cross-feeding by other microbes, which can potentially lead to downstream production of butyrate (41–43). However, in our model, butyrate levels remained unchanged, suggesting this cross-feeding pathway may not have been activated under these specific *in vitro* conditions or within the given timeframe. The study also observed a reduction in acetate and propionate levels in the proximal colon upon probiotic (product P) supplementation within this *in vitro* system. This phenomenon underscores the complex metabolic interactions that can occur in controlled fermentation models and suggests that probiotics may alter microbial metabolite profiles depending on the composition and functionality of the microbial community present (44). This dynamic interplay highlights areas for future investigation regarding how probiotics might drive shifts in microbial ecosystems over time (16, 22, 45, 46).

The combination of Microbiotal M and *L. reuteri* P (synbiotic approach M + P) revealed complementary metabolic patterns within this *in vitro* system. For instance, the co-supplementation M + P showed a numerical trend toward mitigating the acetate reduction observed with probiotic P supplementation alone and resulted in distinct metabolic fingerprints in the distal colon, specifically altered abundance patterns of 1,603 metabolic features (m/z signals) as detected by LA-REIMS analysis, with valid OPLS-DA model differentiation (Q^2^(Y) ≥ 0.5) compared to controls. These preliminary observations suggest that synbiotic formulations warrant further investigation to determine whether they can optimize metabolic outputs and promote balanced microbial ecosystem characterized by enhanced saccharolytic fermentation (increased acetate production) while maintaining controlled proteolytic activity (stable ammonium levels in proximal colon). Synbiotic approaches may therefore represent a promising avenue for future *in vivo* studies aimed at enhancing canine gut health. Moreover, the distinct metabolic patterns observed in different colonic regions *in vitro* reinforce the need for region-specific analysis in future studies addressing gastrointestinal health.

An observed trend toward increased ammonium and branched SCFA levels in the distal colon upon supplementation with any test product warrants careful consideration and represents an important limitation of this pilot study. The accumulation of these proteolytic metabolites has been associated with potentially detrimental effects in the literature (47, 48). The findings suggest that dietary supplements may enhance saccharolytic activity but could also inadvertently promote proteolytic fermentation patterns in specific contexts within in vitro systems. Future in vivo studies should explore strategies to minimize potential proteolytic enhancement through dosage adjustments or the inclusion of compounds that may modulate proteolytic pathways (11). Developing synbiotics formulations that specifically support saccharolytic pathways while controlling proteolytic activity could also represent a critical advancement for future research in this field.

High-resolution metabolomic profiling via LA-REIMS (33, 49–52) revealed distinct and region-specific metabolic shifts in response to dietary interventions in this *in vitro* pilot study. The proximal colon exhibited distinct metabolic fingerprints upon probiotic P and synbiotic M + P treatments, as evidenced by validated OPLS-DA models, including increased lactate production (Figure 6), altered SCFA profiles (reduced acetate/propionate with *L. reuteri* treatment P), and distinct metabolomic signatures. These observations demonstrate the technical capability of the SCIME™ platform to detect metabolic changes in the proximal colon compartment. In contrast, the distal colon’s metabolic response showed distinct patterns with validated OPLS-DA differentiation exclusively upon synbiotic M + P supplementation, while individual treatments did not produce validated metabolic models in this region within this single SCIME™ run. These preliminary observations emphasize the importance of considering regional differences within the colon when designing future dietary intervention studies. Furthermore, the use of advanced metabolomic tools like LA-REIMS demonstrates technical feasibility for providing high-resolution analysis of microbial metabolic activity. A paper published by the present authors in 2024 demonstrated that these same treatments increased beneficial bacteria (*Bifidobacterium, Lactobacillus*) while reducing potentially pathogenic taxa (*Clostridium perfringens, Fusobacterium*), supporting the concept of precision interventions based on specific microbial profiles (23).

These preliminary findings demonstrate the technical feasibility of using the SCIME™ platform for investigating metabolic responses to dietary supplements *in vitro* and suggest potential avenues for future precision nutrition research that could be validated through studies with multiple donors, independent SCIME™ runs, and *in vivo* trials (53). Moreover, this pilot study has demonstrated the capability of the SCIME™ system to detect metabolic changes in response to dietary interventions in a controlled laboratory setting. Future research should expand upon these preliminary observations by incorporating multiple donors, independent experimental replicates, and ultimately *in vivo* validation studies to determine whether similar metabolic modulation occurs in living animals and whether such changes translate to health benefits. Such studies would be valuable in both healthy animals (to understand baseline modulation capacity) and disease-prone or dysbiotic animals, where restoring microbial balance and metabolic function may lead to greater clinical improvements (16, 21, 22, 45, 46).

The preliminary observations from this pilot *in vitro* feasibility study provide technical validation for the SCIME™ platform’s ability to detect metabolic responses to dietary supplements and highlight potential areas for future investigation in canine gastrointestinal health. The observed metabolic patterns *in vitro* suggest that prebiotics, probiotics, and synbiotics may influence fermentation dynamics, SCFA production, and metabolic profiles in ways that warrant further investigation through properly powered studies with multiple donors and independent experimental replicates, followed by *in vivo* validation to assess potential therapeutic relevance in addressing conditions such as dysbiosis, inflammatory bowel disease (IBD), and other chronic gastrointestinal disorders (1). Synbiotics, in particular, showed distinct metabolic fingerprints *in vitro* and represent an interesting target for future research as a potential approach to gut health by combining selective substrate fermentation and microbial modulation. This pilot study demonstrates that dietary supplements can serve as subjects for mechanistic research using advanced *in vitro* models, providing preliminary data to guide the design of future *in vivo* studies in veterinary practice.

Furthermore, this pilot study highlights the utility of advanced *in vitro* models, such as the SCIME™, as technical platforms for preliminary mechanistic research into gut microbiome modulation, reducing the need for animal trials in the initial feasibility assessment stages of product evaluation. A critical and necessary next step would be to validate these preliminary *in vitro* observations through properly designed *in vivo* studies, comparing the metabolic profiles observed here with those measured in the fresh fecal matter of dogs consuming these same supplements. Such validation studies would need to incorporate multiple donors to account for inter-individual variability and determine whether the metabolic patterns observed in this controlled laboratory system are reproducible in living animals.

While this pilot feasibility study provides valuable insights and demonstrates the technical applicability of the SCIME™ platform, several important limitations must be acknowledged:

- The most significant limitation of this study is its reliance on fecal material from a single dog and a single SCIME™ run per treatment condition. All data reflect only this individual dog’s microbiota and cannot be generalized to represent the broader canine population. Biological variability between animals, a critical factor in gut microbiota studies, cannot be accurately assessed. Additionally, microbial composition differs widely even among healthy dogs, and this single-donor approach cannot capture this natural variation. This single-donor design lacks the replication needed to support generalizable conclusions about canine microbiota or treatment effects. In support of this limitation, studies using this canine colonic fermentation platform have enrolled multiple subjects to assess biological variability (25).
- No baseline analyses (e.g., Dysbiosis Index or other microbiome profiling) were performed to confirm that the donor possessed a healthy gut microbiota. While the dog was clinically healthy, with no history of gastrointestinal disorders, no antibiotic exposure within the previous six months, and no diarrheal episodes, clinically normal dogs may still harbor dysbiotic microbiota. This represents a significant limitation that should be addressed in future studies through baseline microbiota characterization of all donors.
- The SCIME™ system is a semi-continuous *in vitro* fermentation model where fecal material from a single donor is maintained in a controlled environment with periodic nutrient addition and removal. In this study, six samples were collected at each time point; however, all six samples were derived from the same SCIME™ run for each treatment, using fecal material from a single dog. This fact represents a critical limitation.
- Therefore, the current data support only descriptive observations and cannot justify claims about treatment effects or population-level conclusions. The *p*-values reported reflect technical variability within a single semi-continuous fermentation system rather than biological variability across individuals, and inferential statistics do not allow generalizations beyond this specific experimental run.
- The use of an *in vitro* model, while sophisticated and enabling long-term controlled experiments, may not fully capture the complexity of *in vivo* conditions. Host-microbiota interactions, immune responses, and host metabolic contributions are not represented in this study. The dosing regimen (daily supplementation directly into the nutrient medium) differs from typical *in vivo* oral administration, where bioavailability, digestion, and stability must be considered, addressing another study limitation.
- Although the single SCIME™ run with the Triple-M-SCIME™ configuration allows for the simultaneous testing of three treatments under identical experimental conditions within one run, providing controlled comparative analysis within the same microbial ecosystem and minimizing technical inter-run variability, multiple independent SCIME™ runs with different donors would be necessary. Future studies should incorporate multiple donors and multiple independent SCIME™ runs to enhance reproducibility and statistical power.
- Finally, the observed trend toward increased proteolytic fermentation (ammonium and branched SCFAs) in the distal colon warrants further investigation in future *in vivo* studies to evaluate whether these *in vitro* patterns translate to *in vivo* conditions.

Incorporating longitudinal studies could provide a more comprehensive picture of microbial dynamics and their long-term implications for host health. Furthermore, exploring the potential for combining synbiotics with other bioactive compounds, such as polyphenols or omega-3 fatty acids, could yield synergistic potential benefits and broaden the scope of dietary management strategies (54).

## 5 Conclusion

This pilot study demonstrates the feasibility of the SCIME™ platform as a tool for preliminary mechanistic research in canine microbiome science, generating initial hypotheses regarding metabolic responses to dietary interventions, particularly to prebiotics, probiotics, and synbiotics. These preliminary findings may pave the way for developing targeted dietary strategies that can improve canine health and well-being. However, definitive conclusions about efficacy, therapeutic applications, or health benefits await future studies with appropriate biological replication and *in vivo* validation. The integration of advanced *in vitro* models and metabolomic technologies represents a promising approach for ethical, controlled preliminary research that can guide the rational design of subsequent *in vivo* investigations in canine gastrointestinal health and disease management.

## Data Availability

The raw data supporting the conclusions of this article will be made available by the authors, without undue reservation.

## Footnotes

^1^ https://wsava.org/global-guidelines/global-nutrition-guidelines/
